# Personal and environmental factors associated with the utilisation of maternity waiting homes in rural Zambia

**DOI:** 10.1186/s12884-017-1317-5

**Published:** 2017-05-04

**Authors:** Cephas Sialubanje, Karlijn Massar, Davidson H. Hamer, Robert A. C. Ruiter

**Affiliations:** 1 Ministry of Health, Monze District Medical Office, P.O. Box 660144, Monze, Zambia; 2 Department of Work and Social Psychology, Maastricht University, P.O. Box 616, 6200MD Maastricht, The Netherlands; 3 Zambia Centre for Applied Health Research and Development, P.O. Box 30910, Lusaka, Zambia; 4 Centre for Global Health and Development Boston University, Crosstown 3rd floor, 801 Massachusetts Avenue, Boston, MA 02118 USA; 5 Department of Global Health, Boston University School of Public Health, Crosstown 3rd floor, 801 Massachusetts Avenue, Boston, MA 02118 USA

**Keywords:** Maternal health, Health seeking behaviour, Maternity waiting home, Kalomo, Zambia

## Abstract

**Background:**

Although the association between the presence of maternity waiting homes (MWHs) and the personal and environmental factors that affect the use of MWHs has been explained in qualitative terms, it has never been tested in quantitative terms. The aim of this study was to test the association between the presence of MWHs and personal and environmental factors that affect the use of MWHs.

**Methods:**

A cross-sectional study was conducted using an interviewer-administered questionnaire from 1^st^ July to 31^st^ August, 2014 among 340 women of reproductive age in 15 rural health centres in Kalomo district, Zambia. Tests of association (chi square, logistic regression analysis, odds ratio) were conducted to determine the strength of the association between the presence of MWHs and personal and environmental factors. Differences between respondents who used MWHs and those who did not were also tested.

**Results:**

Compared to respondents from health centres without MWHs, those from centres with MWHs had higher odds of expressing willingness to use MWHs (adjusted odds ratio [aOR] = 4.58; 95% confidence interval [CI]:1.39–15.17), perceived more benefits from using a MWH (aOR =8.63; 95% CI: 3.13–23.79), perceived more social pressure from important others to use MWH (aOR =27.09; 95% CI: 12.23–60.03) and higher personal risk from pregnancy and childbirth related complications (aOR =11.63; 95% CI: 2.52–53.62). Furthermore, these respondents had higher odds of staying at a health centre before delivery (aOR =1.78; 95% CI: 1.05–3.02), giving birth at a health facility (aOR = 3.36; 95% CI: 1.85–6.12) and receiving care from a skilled birth attendant (aOR =3.24; 95% CI: 1.80–5.84). In contrast, these respondents had lower odds of perceiving barriers regarding the use of MWHs (aOR =0.27; 95% CI: 0.16–0.47). Factors positively associated with the use of MWHs included longer distances to the nearest health centre (*p* = 0.004), higher number of antenatal care (ANC) visits (*p* = 0.001), higher proportions of complications during ANC (*p* = 0.09) and women’s perception of benefits gained from staying in a MWH while waiting for delivery at the health centre (*p* = 0.001).

**Conclusion:**

These findings suggest a need for health interventions that focus on promoting ANC use, raising awareness about the risk and severity of pregnancy complications, promoting family and community support, and mitigating logistical barriers.

## Background

Zambia is one of several low and middle income countries (LMICs) in southern Africa with high proportions of maternal morbidity and mortality [1, 2]. The 2014 Zambia Demographic and Health Survey (DHS) [2] showed that the country has a maternal mortality ratio (MMR) of 398 deaths per 100 000 live births. Most of these deaths are due to known and direct causes which are mostly preventable through use of maternal healthcare services provided by skilled staff in healthcare facilities [2–4].

Nevertheless, low utilisation of antenatal care (ANC), delivery and postnatal care (PNC) services in rural areas of the country has been shown to be one of the main reasons for the high MMR [2–5]. The DHS confirmed this [2]. More than half (53.0%) of the Zambian women do not deliver in health facilities and most (62.0%) do not have access to skilled birth attendants [2, 4, 5]. Moreover, many pregnant women in Zambia delay starting their ANC visits and many do not return for PNC [4, 5].

Studies conducted in Zambia [4–9] and various African countries [10–13] have reported several reasons for the low use of maternal health care services (MHS), such as women’s negative attitudes towards the quality of MHS in health facilities, social and cultural norms, women’s low social status and lack of decision-making autonomy, lack of support from husbands, and negative opinions of influential family and community members about these services (perceived social norms). Other factors include a positive attitude towards traditional birth attendants (TBAs), and physical, financial and logistical barriers such as long distances and high transportation costs. Mitigating these barriers could improve maternal and newborn health outcomes in rural areas of the country [8–10].

To overcome these physical and economic barriers, maternity waiting homes (MWHs) have been established in many low and middle income countries (LMICs), including Zambia [8, 14, 15]. For example, in Kalomo district-the study area for the current study-MWHs have been constructed on ten health centre grounds where women who face various challenges-such as living far from a health centre, lack transport, have high risk pregnancies or a history of complications during the previous pregnancies-can reside and wait for delivery. Those who have no access to MWHs will stay in the ward-usually in the ANC ward [4, 5, 8, 9]. MWHs are “residential facilities, located near a qualified medical establishment, where women living far from the healthcare facility and those with high-risk pregnancies can wait for their delivery and be transferred to a nearby medical facility shortly before delivery, or earlier, should complications arise” [16].

Studies and reviews investigating the effectiveness of MWHs have reported mixed findings [8, 15–21]. For example, a WHO report [16] showed that MWHs are effective in reducing second delay and thus help to detect complications early with effective management. However, there is limited evidence for the effectiveness of the MWHs, and not all studies have reported favourable maternal and newborn health outcomes among women who stayed in MWHs during the last period of their pregnancy [20, 21]. For example, our previous studies [4, 5, 8, 9] showed that, although most health centres provide basic emergency obstetric and newborn care, and that midwives have the skills to identify pregnant women who are at risk while staying in the MWHs as well as those who develop complications during labour and childbirth, the referral system in the district is quite weak. Health centres in the district do not have their own ambulances. They rely on ambulances from the district and mission hospitals for emergency obstetric and newborn care. If a woman in labour develops complications, the midwife will call for an ambulance from the district or mission hospital. However, this usually takes long due to great distances and the poor state of roads. The long distances and poor state of roads usually lead to a second delay preventing pregnant women with complications from accessing maternal and perinatal care provided in the district and other referral hospitals.

Studies from Zambia and other LMICs exploring womens’ perceptions towards MWHs [7–9, 19, 22] show that women had knowledge about MWHs and appreciated the benefits to be gained from using the service. Additionally, husbands and healthcare providers perceived many potential benefits of MWHs [9, 19].

Nevertheless, studies from Zambia [8, 9] and Ghana [23] have reported low utilisation of MWHs. Among the reasons for low utilisation of MWHs reported in the Zambian studies are lack of funds to buy food, baby clothes and cleaning materials for the baby and mother to use during labour at the health centre, concerns about needing someone to remain at home and take care of the children and husband, and concerns about the deplorable state of the MWHs. Similarly, the study from Ghana [23] reported various reasons for the low use of MWHs-such as costs associated with staying in a MWH, the hardship of staying away from home and the absence of health personnel in healthcare facilities.

Despite providing important insight into both the perceived benefits of using MWHs as well as the reasons for poor utilisation of these services, these studies were exploratory and qualitative in design. Thus, although, the association between the presence of MWHs and the personal and environmental factors that affect their use has been explained in qualitative terms, this association has never been tested using quantitative methods. Moreover, it is not known whether the strength of these associations differs between the respondents who have access to health centres with a MWH and those who do not. The aim of the current study, therefore, was to test the strength of the association between the presence of MWHs and personal and environmental factors which affect the use of MWHs among respondents with and without access to MWHs. Moreover, the study aimed to compare the women who had used the MWHs and those who did not. Insight into these associations and factors is important for the design of public health interventions for improving utilisation of MWHs in rural Zambia.

### Theoretical framework

The present study used Fishbein and Ajzen’s Reasoned Action Approach (RAA) [24] and the Health Belief Model [25, 26] as the theoretical framework. For details on the Reasoned Action Approach and the Health Belief Model and their application, see Sialubanje et al. [5].

## Methods

### Study design and setting

The study used cross-sectional design, and data were collected from 15 health centre catchment areas of Kalomo district, Zambia [27–29]. For details on Kalomo district profile see Sialubanje et al., [4, 5, 8, 9].

### Study population

The study participants were sampled from women of childbearing age (mean age 25.60 years, SD = 6.85). Of these, 203 women (mean age = 24.69 years, SD = 6.61) were recruited from health facilities with a MWH, and 137 women (mean age = 26.94, SD = 7.01) from health facilities without a MWH. To be eligible to participate in the interview, women must have delivered in the past 12 months prior to the survey and resided in the area for more than six months.

### Sampling techniques

The study utilised a multi-stage convenience sampling method. All ten health centres with a MWH in the district were identified and included in the study, after which, five out of a total 25 health centres without a MWH were purposefully selected and included in the study. Fourteen villages from the fourteen rural health centres and one compound from the semi-urban health centre were randomly sampled. Since there was more than one village in each health centre catchment area, one village was purposively selected based on accessibility and advice from community health workers and headmen. The number of respondents surveyed from each village was evenly distributed. However, due to a lack of information and the unstructured nature of housing units in the area, it was not possible to select respondents using systematic sampling methods.

### Ethical considerations

The Tropical Disease Research Centre Ethics Review Committee and the Ministry of Health Research and Ethics Committee in Zambia provided the ethical approval for the study (study number TDRC/ERC/2OO5/29/12).

Before starting the survey, research assistants read out the aims of the study to the participants. They also explained that the respondents’ names would not be written on the questionnaire or on the informed consent form. Moreover, respondents were informed that survey participation was voluntary, that they would not receive any direct benefits from the study, and that they were free to discontinue the survey at any point if they felt uncomfortable. Participants were informed that the purpose of the survey was to collect information on what they thought affected their use of MWHs, and that the information would be used to inform and guide future government policies on MWHs. Written informed consent was obtained by having the participants either sign the consent form or mark with an ‘X’. Respondents who were able to write were made to sign on the consent form, whereas those who could not write were made to mark with an ‘X’.

### Data collection

Two trained research assistants who were supervised by the principal investigator collected the data. The research assistants were recruited from within Kalomo district and were both female, aged 22 and 25 respectively, and had a full grade 12 certificate. The research assistants received a one day face-to-face training on the study and the questionnaire. Female research assistants were preferred to male for cultural reasons and in order to ensure optimal interaction with the mothers. Moreover, to minimise information concealment from the respondents during the survey, research assistants spoke both English (the official language) and Tonga, the local language.

A week before the survey, women were informed about the survey date by the village headmen and neighbourhood health committee (NHC) members. On the agreed day, the principal investigator and the research assistants travelled to the respective health centres from which the research assistants were directed into the households by the NHC members and community volunteers. Because of high illiteracy levels in the area, the questionnaire was translated into Tonga. Women who were able to read were allowed to go through the questionnaire by themselves; the interviewer merely confirmed whether the questionnaire was correctly and completely answered. All the interviews took place in the participant’s home or at a nearby convenient place-normally a quiet place under a tree, a few meters from the participant’s house. Each survey lasted between thirty to forty minutes.

### Measures

The questionnaire was developed by the research team based on variables described by social cognitive theories of human behaviour, including the theory of Reasoned Action Approach [24] and the Health Belief Model [25] as well as findings from our previous studies in the area [4, 8, 9]. The research instrument was first developed in English, translated to Tonga by an independent bi-lingual expert, and then back-translated to English. The final version of the questionnaire was both in Tonga and English (see supplementary file for the English version of the questionnaire). All items were answered on a 5-point Likert scale ranging from 1 = fully disagree to 5 = fully agree, or similar labels.

We used factor analysis to check which items, based on theory, should measure a particular psychosocial construct combined into one factor or not. Items that showed strong internal consistency (Cronbach’s alpha > 0.6 or r > 0.40) were combined and averaged into one index. If items did not combine into one index, factor analyses were conducted using principal axis factoring as an extraction method, and an oblimin rotation. After inspection of the scree plot (that is, a plot which displays the eigenvalues associated with a component or factor in descending order versus the number of the component or factor), sum measures were created with Eigenvalue score of 1.0 or higher and included those items that had factor loadings of 0.4 or higher. See Table 1 for the different items used in the present study and how they were clustered to measure underlying psychosocial constructs.

**Table 1:** Factor analysis

Predictor	Item	N items/Cronbach’s alpha(α)	Mean (SD)
Cognitive attitude	I consider a clinic with a mothers’ shelter more *beneficial* to the mother and baby than a clinic without a mother’s shelter.	17 items; α = .75	4.28(0.06)
	Staying in the mother’ shelter is *important* because it prevents women (who live far from the clinic) from giving birth at home or on the way to the clinic		
	Staying in the mother’ shelters is *helpful* as it makes it *easier* for women who live far from the clinic to give birth at the health centre		
	Staying in the mother’s shelters will *help* mothers receive assistance from a skilled birth attendant (nurse, midwife) during labour		
	Staying in the mothers’ shelter will help prevent labour complications for the baby and mother who live far from the clinic		
	I would rather deliver at a clinic with a mother’s shelter than one without a mother’s shelter		
	Going early to the mother’s shelter to wait for delivery from there is *wiser* than waiting for delivery at home until the woman is in established labour		
	Staying in the mothers’ shelter at the clinic is beneficial to the mother and baby as it would help them receive care from the midwife/nurse during labour and thus reduce complications		
	Waiting at the mother’s shelter will help women find assistance from the nurses and midwives if they develop labour complications		
	Waiting for delivery at the mothers’ shelter prevents pregnant women from reaching the clinic late due to long distances and lack of transport		
	It is *important* for husbands and other family members to allow their wives to wait for labour in the mothers’ shelter if they live far from the clinic		
	It is wise for the husbands who live far from the clinic to allow their wives to wait for delivery in the mothers’ shelters as they know their wives will be safe there		
	Compared to a clinic without a mothers’ shelter, a clinic with a mothers’ shelter would make it easier for me to wait for delivery from the clinic		
	Compared to a clinic without a mothers’ shelter, a clinic with a mothers’ shelter would make it easier for me to give birth at the clinic		
	Compared to a clinic without a mothers’ shelter, a clinic with a mothers’ shelter would make prevention of complications to the mother and baby *more feasible* as accompanying relatives can easily walk to the clinic to inform midwives and other clinic staff in case of complications		
	Compared to a clinic without a mothers’ shelter, a clinic with a mothers’ shelter would make prevention of complications to the mother and baby *more feasible* as the mother can easily walk to the clinic to get help from the midwives and other clinic staff in case of problems		
Affective Attitude	Stay in the mothers’ shelter while waiting for delivery will be *enjoyable* to the women as she will rest	7 items; α = .72	3.52 (0.54)
	Staying at the mothers’ shelter while waiting for delivery *hurts* the pregnant woman as it separates her from her husband and children		
	Staying in the mother’s shelter while waiting for delivery will be *safe*		
	Sleeping conditions at the clinic with a mothers’ shelter will be *pleasant*		
	Mothers’ shelters will provide more *enjoyable* bathing and washing conditions for the pregnant mothers		
	Mothers’ shelters would provide more *satisfactory* cooking facilities for the pregnant women and their relatives		
	Waiting for delivery at the mothers’ shelters will be *stressful* to the mother due to lack of money for food and other requirements		
	Staying in the mothers’ shelters will *hurt* most pregnant women as they have no one to take care of them while waiting for labour.		
Descriptive Norms	Many women in my community who live far from the clinic prefer giving birth at a clinic with a mothers’ shelter (if they are present)	13 items; α = .60	3.69 (0.16)
	Many women in my community who live far from the clinic prefer clinic with a mothers’ shelter (if they are present) rather than those without a mothers’ shelter		
	Many women in my community depend on other family members to decide for them on whether they should go and stay in the mothers’ shelter or not		
	Many women are happy with other people in the family and community deciding for them on whether they should go and stay in the mothers’ shelter or		
	In my community, husbands are the ones who decide on whether the woman should go and stay in the mothers’ shelter or not		
	In my community parents and mothers-in-law are the ones who decide on whether the woman should deliver at the clinic or not		
	Many husbands allow their wives to leave home to go and deliver at the clinic		
	In my community many husbands provide resources for their wives to use while waiting for labour at the mothers’ shelter		
Injunctive Norms	Many people in my community believe that mothers’ shelters are beneficial to the mothers who live far from the clinic as they can go and stay there and wait for labour	9 items; α = .82	4.90 (0.20)
	Many women in my community believe that, to avoid labour complications, women should go and stay in the mothers’ shelter as they wait for labour		
	If I live far from the clinic, my husband would approve of my delivering at a clinic with a mothers’ shelter rather than at the clinic with no shelter.		
	My husband would be more likely to approve of my delivering at a clinic with a mother shelter than at a clinic without a mothers’ shelter		
	My husband would approve of my leaving home to go and stay in the mothers’ shelter as I wait for delivery at the clinic		
	My children would approve of my leaving home to go and stay in the mothers’ shelter as I wait for delivery at the clinic		
	My parents and mother- in-law would approve of my leaving home to go and stay in the mothers’ shelter as I wait for delivery at the clinic		
	TBAs in my community would approve of my leaving home early to go and stay in the mothers’ helter as I wait for labour at the clinic		
	Important people in my community including the headmen approve of my leaving home to go and wait for delivery at the clinic		
	Important people in my community including nurses, neighbourhood health committee members (NHCs) and Community Health workers (CHWs) would want me to leave home to go and stay in the mothers’ shelter as I wait for labour at the clinic		
	I want to do what my husband, children and other family members want and stay in the mothers’ shelter while waiting for labour		
Perceived Behavioural Control	The desire to have the baby examined would make it easier for me to stay for a few days (hours) after delivering in order for me and the baby to be examined by the midwives/nurses at the clinic	15 items; a = .60	4.24 (0.08)
	I am confident I can convince my husband to allow me to leave home early enough to go and stay in the mothers’ shelter as I wait for labour at the clinic		
	I am confident that I can go and stay at the mothers’ shelter to wait for delivery even if other people in my community might have negative opinions about staying in the mothers’ shelters		
	I am confident I can go and stay at the mothers’ shelter even if I don’t have things to use (like baby clothes, jik, etc.)		
	Being accompanied by a relative would make it easier for me to stay in the mothers’ shelter as I wait for labour at the clinic		
	I am confident I can leave home early enough to go and stay in the mothers’ shelter as I wait for labour at the clinic		
	Provision of beddings to the mothers by the clinic staff would make my stay in the mothers’ shelter more *manageable* as I wait for labour at the clinic		
	Provision of food to the mothers would make my stay in the mothers’ shelter more *feasible*		
	Regular visits by midwives and other clinic staff to see if I need help would make my stay at the mothers’ shelter *more feasible*		
Risk Perception	I believe pregnancy complications (i.e., bleeding, pre-eclampsia) are very severe	5 items; α = .83	4.98 (0.10)
	I believe labour complications such as obstructed labour, bleeding and, pre-eclampsia are very severe		
	I believe that complications require a skilled attendant’ assistance.		
	I believe that, all pregnant women (regardless of age, number of children and history of past delivery) are at risk of these complications		
	I believe that I am personally at risk of developing complications during pregnancy and labour		
Perceived Barriers	Concerns about my husband refusing to allow me to go and stay at the mothers’	14 items; α = .70	2.37 (0.17)
	Concerns about who to leave my children with		
	Concerns about my marriage and husband when I am away		
	Concerns about money for food, baby clothes and other requirements (like jik, plastic paper)		
	Concerns about people to accompany and stay with me in the mothers’ shelter		
	Concerns about transport to the clinic and going back home after delivering at the clinic		
	Concerns about sleeping conditions in (including space) in the mothers’ shelters		
	Concerns about availability of blankets in the mothers’ shelter		
	Concerns about availability of nurses and midwifery to assist me in case of pregnancy or labour complications like breeding, high blood pressure, fitting) during my stay in the mothers’ shelters		
	Concerns about lack of privacy in the labour ward when I go into labour		

Intention was measured using one item: “If I am pregnant again and due for labour, I will make efforts to go and stay at the maternity waiting home as I wait for labour at the clinic”.

In total, 25 items were constructed to measure attitude (table 1). Factor and reliability analyses revealed two underlying variables: *cognitive attitude towards MWHs (17* items, α = 0.75). The other attitude variable was *affective attitude toward staying in a MWH* (7 items, α = 0.72*).* Similarly, factor and reliability analyses were performed on the 22 items measuring perceived social norms, which resulted in two variables: one of these was *descriptive social norms* towards MWH use (13 items, α = 0.60), and injunctive *social norms* towards MWHs (9 items, α = 0.82). Seventeen items were constructed to measure perceived behavioural control (PBC), and factor and reliability analyses resulted in one variable (15 items, α = 0.60). The five items measuring risk perception were also averaged into one variable (with five items, α = 0.83). Finally, perceived barriers towards using MWHs were measured using seventeen items. Factor analysis revealed one variable (14 items, α = 0.70).

### Data analysis

Descriptive statistics were used to compute percentages of respondents’ demographic and past maternal health seeking behaviour. After inspection of the data and descriptive analysis, we noticed that the data were severely negatively skewed and the assumption of normality was violated. We performed a median split procedure on the psychosocial measures-such that scores including the median and below were dummy-coded as 0 (representing low to moderate scores); and scores above the median were dummy-coded as 1 (representing high scores). To investigate the univariate association between psychosocial measures and intention to use a MWH, and to compare scores on psychosocial measures, sociodemographic variables and past behaviour between those with and those without access to MWHs, Chi-square tests and logistic regression analyses were used. Crude odds ratios (ORs) with 95% confidence intervals (CI) were computed to estimate the effect size. Furthermore, independent t-tests and Cohen’s d [30] were used to investigate whether the respondents from the two groups differed with regard to sociodemographic and economic factors (age, number of children, and distance to the nearest health centre). Finally, adjusted odds ratios (aOR) were calculated to control for confounding due to age, parity, and distance to the nearest health centre (*p* < 0.05).

## Results

Table 2 summarizes the demographic characteristics of the 340 respondents who participated in the survey as well as differences between the respondents with and without access to MWHs. Respondents from health centres with MWHs were significantly younger (*p* = 0.002), had fewer children (*p* = .004), and lived closer to the health centres (*p* = 0.005). Moreover, there were significant differences between the two groups with regard to marital status (*p* = 0.004) and income level (*p* = 0.001). There was no significant difference with regard to level of education (*p* = 0.097).

**Table 2:** Sociodemographic characteristics of the respondents (*n* = 340)

Variable				*P* value
	Total Sample (*N* = 340)	MWH (*n* = 203)	No MWH (*n* = 137)	
Age (M, SD)	25.54 (6.96)	24.60 (6.78)	26.94 (7.01)	.002
Children (M, SD)	3.05 (2.08)	2.78 (1.02)	3.45 (2.34)	.004
Marital status (N, %)				
Single	45 (13.2%)	37 (18.2%)	8 (5.8%)	.004
Married	287 (84.4%)	160 (78.8%)	127 (92.7%)	
Separated	1 (0.3%)	1 (0.5%)	0 (0%)	
Divorced	3 (0.9%)	1 (0.5%)	2 (1.5%)	
Widow	4 (1.2%)	4 (2.0%)	0 (0%)	
Education Level (N, %)				
Never Attended	9 (2.6%)	6 (3.0%)	3 (2.2%)	.097
Grade 1–4	31 (9.1%)	15 (7.4	16 (11.7%)	
Grade 5–7	127 (37.4%)	70 (34.4	57 (41.6%)	
Grade 8–9	133 (39.1%)	91 (44.8%	42 (30.7%)	
Grade 10–12	35 (10.3%)	20 (9.9%)	15 (10.9%)	
Higher education	5 (1.5%)	1 (0.5%)	4 (2.9%)	
Occupation (N, %)				
Dependant	42 (12.4%)	37 (18.2%)	5 (3.6%)	<.001
House wife	81 (23.8%)	56 (27.6%)	25 (18.2%)	
Farmer	130 (38.2%)	68 (33.3%)	62 (45.3%)	
Business woman	57 (16.8%)	29 (14.3%)	28 (20.4%)	
Formal Employment	30 (8.8%)	1 3 (6.4%)	17 (12.4%)	
Income Level per month in Zambian Kwacha* (N, %)				
<100	148 (43.5%)	113 (55.7%)	35 (25.5%)	<.001
100–249	54 (15.9%)	23 (11.3%)	31 (22.6%)	
250–499	58 (17.1%)	32 (15.3%)	26 (19.19%)	
500–999	35 (10.3%)	19 (9.4%)	16 (11.7%)	
>1000	45 (13.2%)	16 (7.9%)	29 (21.2%)	
Distance to the nearest health centre in kilometres (M, SD)	12.31 (10.40)	11.01 (9.50)	14.24 (11.37)	.005

*One US dollar is equivalent to 10 Zambian Kwacha

Table 3 summarizes the respondents’ past health seeking behaviour as well as the differences between the respondents with and those without access to MWHs. There were significant differences between the respondents with and those without access to MWHs with regard to place of delivery (*p* < 0.001), duration of stay at the health centre before delivery (*p* < 0.04) and birth attendant (*p* < 0.001). Additionally, there was a trend towards a difference between the two groups with regard to ANC use (*p* < 0.06). In contrast, there were no significant differences between the two groups with regard to history of complications during pregnancy (*p* = 0.27) or delivery (*p* = 0.60).

**Table 3:** Utilisation of maternal health services during previous pregnancy

Variable	Mean (SD)/n (%)			*P* value
	Total sample (*N* = 340)	MWHs (*N* = 203)	No MWHs (*N* = 137)	
ANC use (%)				
Yes	339 (99.7%)	203 (100%)	136 (99.3%)	.06
No	1 (0.3%)	0 (0%)	1 (0.7%)	
ANC booking in months/gestational age (M, SD)	4.33 (1.33)	4.34 (1.26)	4.32 (1.42)	.10
Number of ANC visits (M,SD)	3.76 (1.01)	3.76 (0.99)	3.74 (1.04)	.96
Any complications during pregnancy and ANC (N, %)				
Yes	80 (23.5%)	52 (25.6%)	28 (20.4%)	.27
No	260 (76.5%)	151 (74.4%)	109 (79.6%)	
Type of complications during pregnancy (N, %)				
PV bleeding	13 (3.8%)	10 (4.9%)	3 (2.2%)	.60
Abdominal pain	36 (10.6%)	21 (10.3%)	15 (10.9%)	
Abnormal lie	7 (2.1%)	5 (2.5%)	2 (1.5%)	
High Blood Pressure	15 (4.4%)	9 (4.4%)	6 (4.4%)	
High blood sugar level	1 (0.3%)	1 (0.5%)	0 (0%)	
Baby not growing well	3 (0.9%)	2 (1.0%)	1 (0.7%)	
Baby dead in the uterus	5 (1.5%)	5 (2.5%)	0 (%)	
No complication	260 (76.5%)	151 (74.4%)	110 (80.3%)	
Place of delivery (N, %)				
Home	57 (16.8%)	19 (9.3%)	38 (27.7%)	<.001
Health Centre or hospital	271 (79.7%)	179 (88.2%)	92 (67.2%)	
On the way	12 (3.5%)	5 (2.5%)	7 (5.1%)	
MWH present at health centre of delivery (N, %)				
Yes	251 (78.8%)	173 (85.2%)	78 (56.9%)	<.001
No	20 (5.95%)	6 (1.8%)	14 (4.1%)	
Delivered at home or on the way to the health centre	69 (20.3%)	24 (7.1%)	45 (13.2%)	
Stay at the health centre before delivery (N, %)				
Yes	98 (28.8%)	66 (32.5%)	32 (23.4%)	.04
No	242 (71.2)%	137 (67.5%)	105 (76.6%)	
Place of stay before delivery (N, %)				
Ward	20 (5.9%)	10 (4.9%)	10 (7.3%)	.02
MWH	78 (22.9%)	56 (27.6%)	22 (16.1%)	
Stayed at home or delivered on the way	242 (71.2%)	137 (67.5%)	105 (76.6%)	
Reasons for staying at the health centre before delivery (N, %)				
Long distances	17 (5%)	6 (3.0%)	11 (8.0%)	.006
Lack of transport	1 (0.3%)	0(0.00%)	1 (0.7%)	
Fear of complications	49 (14.4%)	38 (18.7%)	11 (8.0%)	
History of complications	4 (1.2%)	2 (1.0%)	2 (1.5%)	
Advised by nurse	22 (6.5%)	18 (8.9%)	4 (2.9%)	
Just decided to stay	6 (1.7%)	2 (1.0%)	3 (2.2%)	
Stayed at home or delivered on the way	242 (71.2%)	137 (67.5%)	105 (76.6%)	
Birth attendant (N, %)				
Doctor	33 (9.7%)	22 (10.8%)	11 (8.0%)	<.001
Clinical Officer	13 (3.8%)	11 (5.4%)	2 (1.5%)	
Nurse	222 (65.3%)	144 (70.9%)	78 (56.9%)	
TBAs	18 (5.3%)	9 (4.4)	9 (6.6%)	
Family member/neighbour	54 (15.9%)	17 (8.4%) 7 (3.4)	37 (27.0%)	
Duration of stay at the health centre before delivery (M, SD)		9.06 (10.93)	4.91 (6.74)	.02
Complications during or after delivery				
Yes	70 (20.6%)	48 (23.6%)	22 (16.1%)	.09
No	270 (79.4%)	155 (76.4%)	115 (83.9%)	
Type of complication during or after delivery (N, %)				
Bleeding	41 (12.1%)	29 (14.3%)	12 (8.8%)	.44 .44
Retained placenta	2 (0.6%)	1 (0.5%)	1 (0.7%)	
High Blood Pressure	2 (0.6%)	2 (1.0%)	0 (0.0%)	
Fitting	3 (0.9%)	1 (0.5%)	2 (1.5%)	
Obstructed labour	13 (3.8%)	7 (3.4%)	6 (4.4%)	
Ruptured uterus	1 (0.3%)	1 (0.5%)	0 (0.0%)	
Caesarian section	5 (1.5%)	4 (2.0%)	1 (0.7%)	
Baby died	2 (0.6%)	2 (1.0%)	0 (0.0%)	
Had no complications	270 (79.4%)	155 (76.4%)	115 (83.9%)	
Duration of stay at the health centre after delivery in hours (M, SD)	13.60 (20.29)	14.31 (21.50)	12.18 (17.66)	.58
Age of child at first PNC visit in days (M, SD)	7.43 (4.90)	7.13 (4.80)	7.90 (5.05)	.17

Since our preliminary analyses showed differences between respondents from health centres with MWHs versus those without MWHs with regard to age, parity, marital status, level of income and distance to the nearest health centre, we controlled for these variables in our analysis by computing the aOR (Table 4).

**Table 4:** Adjusted odds Ratios for the association between the presence of a MWH and use a MWH

Variable	Mean (SD)/n(%)		Adjusted OR	95% CI		*P*-value
	MWH	No MWH		Lower Bound	Upper Bound	
Intention	199 (98%)	124 (90.5%)	4.58	1.39	15.17	.013
Cognitive Attitude	198 (97.5%)	110 (80.3%)	8.63	3.13	23.79	<.001
Affective Attitude	79 (38.9%)	23 (16.9%)	4.20	2.33	7.57	<.001
Descriptive Norms	161 (79.3%)	119 (86.9%)	0.49	0.26	0.94	.03
Injunctive Norms	194 (95.6%)	68 (49.6%)	27.09	12.23	60.03	<.001
PBC	95 (46.8%)	56 (40.9%)	1.06	0.66	1.69	ns
Risk Perception	201 (99%)	122 (89.1%)	11.63	2.52	53.62	.002
Perceived Barriers	58 (28.6%)	88 (64.2%)	0.27	0.16	0.47	<.001
Complications during ANC	52 (25.6%)	28 (20.4%)	1.38	0.79	2.4	ns
Stay at the clinic	68 (33.5%)	32 (23.4%)	1.78	1.05	3.02	.03
Stay at the MWH	56 (27.6%)	23 (16.8%)	2.30	0.71	7.39	ns
Place of delivery	179 (88.2%)	92 (67.2%)	3.36	1.85	6.12	<.001
Birth attendant	177 (87.2%)	91 (66.4%)	3.24	1.80	5.84	<.001
Complications during labour	48 (23.6%)	22 (16.1%)	1.75	0.96	3.19	ns

Odds Ratio adjusted for age, parity, marital status, level of income, and distance to the nearest health centre

Logistic regression showed that, compared to respondents from health centres without MWHs, those from centres with MWHs were more likely to express high intention to use MWHs (aOR = 4.58; 95% CI: 1.39–15.17), perceive benefits from using a MWH (aOR = 8.63; 95% CI: 3.13–23.79), perceive pressure from important others regarding MWH use (aOR = 27.09; 95% CI :12.23–60.03), and perceive risks regarding pregnancy and childbirth related complications (aOR = 11.63; 95% CI:2.52–53.62). Moreover, regarding the respondents who went to stay at the health centre to wait for labour due to high risk pregnancies or long distances from home, those from the health centres with MWHs had higher odds of staying at the health centre to wait for labour there (aOR = 1.78 1,78; 95% CI:1.05–3.02). In contrast, there was no an association between the presence of a MWH and the likelihood of a woman developing complications during and after labour (aOR = 1.75; 95% CI: 0.96–3.19). Similarly, there were no significant associations between the presence of MWH and PBC or with having stayed at a MWH during a previous pregnancy.

We conducted additional analyses (using independent t-tests and chi square tests) to investigate the difference between the respondents who used MWHs and those who did not. Since some health facilities did not have MWHs, we included only the 98 respondents who stayed at the health facility to wait for labour (that is, those who stayed in the ward and those who stayed in the MWHs). We found that respondents who used MWHs covered significantly longer distances from their place of residence to the nearest health facility and had completed more ANC visits (*p* < 0.001). These respondents also perceived more benefits from staying at the health facility to await delivery (*p* = 0.001). There were no significant differences between the two groups with regard to the proportion of complications detected during ANC (*p* = 0.09), age, parity, marital status, occupation, level of education or income. In addition, there were no differences with regard to perceived benefits from MWH use (Table 5).

**Table 5:** Characteristics of the respondents who used a MWH and those who did not

Variable	Mean (SD/n(%)			*P* value
	Total Sample (*N* = 340)	Users (*N* = 98)	Non users (*N* = 242)	
Age in year (M, SD)	25.54 (6.96)	25.80 (6.70)	25.44 (7.07)	.73
Children (M, SD)	3.05 (2.08)	3.18 (2.34)	3 (1.97)	.49
Marital status (N, %)				
Single	45 (13.2%)	13 (13.3%)	32 (13.2%)	.50
Married	287 (84.4%)	81 (82.7%)	206 (85.1%)	
Separated	1 (0.3%)	1 (1.0%0	0 (0%)	
Divorced	3 (0.9%)	1 (1.0%)	2 (0.8%)	
Widow	4 (1.2%)	2 (2.0%)	2 (0.8%)	
Education Level (N, %)				
Never Attended	9 (2.6%)	5 (5.15)	4 (1.7%)	.60
Grade 1–4	31 (9.1%)	9 (9.2%)	22 (9.1%)	
Grade 5–7	127 (37.4%)	33 (33.7%)	94 (38.8%)	
Grade 8–9	133 (39.1%)	38 (38.8%)	95 (39.3%)	
Grade 10–12	35 (10.3%)	12 (12.2%)	23 (9.5%)	
Higher Education	5 (1.5%)	1 (0.5%)	4 (2.9%)	
Occupation (N, %)				
Dependant	42 (12.4%)	10 (10.2%)	32 (13.2%)	.23
House wife	81 (23.8%)	20 (20.4%)	61 (25.2%)	
Farmer	130 (38.2%)	45 (45.9%)	85 (35.1%)	
Business woman	57 (16.8%)	17 (17.3%)	40 (16.5%)	
Formal Employment	30 (8.8%)	6 (6.1%)	24 (9.9%)	
Income Level (N, %)				
<100	148 (43.5%)	46 (46.9%)	102 (42.1%)	.36
100–249	54 (15.9%)	14 (14.3%)	40 (16.5%)	
250–499	58 (17.1%)	20 (20.4%)	38 (15.7%)	
500–999	35 (10.3%)	10 (10.2%)	25 (10.3%)	
>1000	45 (13.2%)	8 (8.2%)	37 (15.3%)	
Distance to the nearest clinic in kilometres (M, SD)	12.31 (10.40)	14.94 (10.50)	11.25 (10.19)	.004
ANC use				
ANC booking in months/gestational age (M, SD)	4.33 (1.33)	4.15 (1.29)	4.40 (1.33)	.11
Number of ANC visits (M, SD)	3.76 (1.01)	4.05 (1.13)	3.64 (0.93)	.001
Complications during pregnancy and ANC (N,%)				
Yes	80 (23.5%)	29 (29.6%)	51 (21.1%)	.09
No	260 (76.5%)	69 (70.4%)	191 (78.9%)	
Psychosocial variables (M,SD)				
Intention	4.95 (0.24)	4.98 (0.20)	4.93 (0.25)	.08
Cognitive Attitude	4.98 (0.07)	4.99 (0.04)	4.98 (0.08)	.25
Affective Attitude	4.93 (0.22)	4.98 (0.08)	4.91 (0.25)	<.001
Descriptive Norms	4.91 (0.27)	4.93 (0.27)	4.90 (0.26)	.34
Injunctive Norms	4.92 (0.16)	4.94 (0.15)	4.91 (0.17)	.19
PBC	4.90 (0.13)	4.91 (0.12)	4.90 (0.13)	.75
Risk Perception	4.98 (0.10)	4.99 (0.05)	4.98 (0.11)	.35
Perceived Barriers	1.15 (0.15)	1.15 (0.14)	1.15 (0.16)	.82

## Discussion

Consistent with our previous qualitative findings [4, 8, 9], our results show that, compared to the respondents who used health centres without a MWH, those who used health centres with a MWH were more likely to express a higher behavioural intention to use a MWH and perceived more benefits from using a MWH.

These findings are consistent with previous qualitative studies [4, 8, 9, 31] that revealed that women in rural Zambia were aware of the MWHs, and pregnant women perceived benefits from using the service. Interestingly, the current study indicates that, if a MWH is present at a healthcare facility, women’s attitude towards MWHs as well as their behavioural intention to use it are more positive. This finding suggests that the presence of MWHs might improve utilisation of the service, especially for women residing far from the healthcare facilities and those with high-risk pregnancies. This is in line with the original WHO intention for MWHs to improve access to facility-based skilled birth attendance [16].

Moreover, in line with our qualitative findings [4, 8, 9, 31] the current findings suggest that women who have access to health centres with MWHs were significantly more likely to perceive social pressure (injunctive norms) from influential family and community members-such as husbands, parents and friends-to use or not to use MWHs than women without access to MWHs. In contrast, our current findings show that sociodemographic variables such as age, number of children, marital status, and distance to the nearest health centre did not influence this association. This finding contradicts our previous studies [4, 8, 9, 31] which suggested that older women with many children were more likely to perceive and comply with social pressure from important others to use or not to use a MWH. The explanation for this finding could be that the mere presence of a MWH in the community and the respondents’ observation of important others’ use of the service-together with the health messages they receive at the health centre during ANC-might increase injunctive norms. This could lead respondents from the health centres with MWHs to perceive more pressure to use the service compared to their counterparts from health centres without MWHs. Future research could focus on determining the source of the perception of injunctive norms in favour of using an intervention such as the MWH, as well as investigating the effect of the health promotion programmes on the perception of these norms.

Regarding women’s perceived risk of developing pregnancy and labour complications and their severity, our findings show that, irrespective of whether a MWH was present or not, most respondents believed that they were personally at risk of developing pregnancy and labour complications. Further, all the respondents believed that these complications were very serious, both to the pregnant woman and to the unborn or newborn baby. Interestingly, the current findings show that respondents perceived their risk differently, depending on whether they lived close to a health centre with a MWH or not. For example, the odds of personal risk perception for pregnancy and childbirth related complications among the respondents with access to a MWH were more than ten times higher than those of respondents without access to a MWH. This effect remained even after controlling for possible confounding variables such as distance to the nearest health facility. This finding suggests the unique role MWHs play in enhancing women’s risk perception regarding the severity and personal susceptibility to pregnancy and childbirth-related complications. Again, one might hypothesise that increased health promotion efforts in the facilities with a MWH could lead to the increased risk perception. However, the wide range of the 95% CI and the cross-sectional design of our study preclude drawing firm conclusions. Further research-preferably randomised control trials-is needed to confirm this association.

Interestingly, the current findings show that, despite having a high risk perception and perceiving benefits from using MWHs, many women did not use the service due to various barriers. For example, our findings show that less than one quarter (23.8%) of the respondents stayed at the MWH before delivery. Moreover, the odds of perceiving barriers that prevented use MWH were almost four times higher among women who were surveyed near a health centre without a MWH than those from health centres with MWHs. The main barriers reported by these respondents included concerns about travelling long distances to health centres with a MWH, as well as insufficient funds to buy food and other requirements for the mother and baby. Other barriers included a lack of decision-making autonomy and dependence on the husband and other family members-such as parents-for decision-making, and concerns about leaving their husbands and children at home when they were away at the MWH. Furthermore, these respondents were concerned about the deplorable state of the MWHs [8, 9, 31]. This finding is consistent with our previous qualitative findings [8, 9, 31] as well as those from other LMICs. For example, a study in Ghana [23] reported low utilisation of MWHs and highlighted important contextual barriers affecting utilisation of MWHs. Together, these studies show that expressing a higher behavioural intention to use a MWH or perceiving more benefits from using the service do not necessarily mean that women will actually go there to stay and wait for delivery.

This finding is also consistent with previous studies which have all reported the challenge of the “intention-behaviour gap” and highlighted the complexity of health seeking behaviour [32–36]. Thus, these findings suggest a need for public health interventions to not only focus on the target populations’ attitude and intention, but to also target factors that might make it difficult for women to enact the intended behaviour [3, 36, 37]. However, to determine causal relationships, future studies should be longitudinal and experimental in design.

Despite the reported low utilisation of MWHs, our findings suggest that the presence of a MWH might positively affect pregnant women’s use of healthcare services by reducing the second phase delay in health seeking behaviour. For example, women who were surveyed at health centres with MWHs were more likely to go and stay at the health centre and wait for labour from there, were more likely to give birth at a health centre and were more likely to receive care from a skilled birth attendant. Moreover, these findings suggest that these women had higher odds of developing complications than those without access to MWHs. This could be due to the fact that MWHs lead to a reduction in second phase delay, thus leading to more early detection and management of complications which can result in better maternal and perinatal health outcomes. This finding is consistent with our previous studies that reported that MWHs might play an important role in reducing labour complications and improving maternal and newborn health outcomes [8, 9, 14–18, 29–31].

Thus, although establishment of MWHs in rural areas could lead to an increase in access to and utilisation of healthcare facilities and skilled birth attendance [7–23], we cannot draw conclusions on the role of MWHs in improving maternal and newborn health outcomes. Moreover, the available evidence on the direct role of MWHs in improving maternal and newborn health outcomes is inconclusive [16–21]. Further research is needed to establish this relationship.

Finally, our findings showed that respondents who stayed in the MWHs to wait for labour significantly differed from those who did not. For example, respondents who left home to go and stay at the health facility to wait for labour lived significantly further away from the health centres than those who did not. These respondents also reported a significantly higher number of ANC visits, more complications detected during ANC visits and perceived more benefits from staying at the health facility for labour. These findings are in line with the original idea of the MWHs, which is to mitigate long distances to the healthcare facilities for the women who live in rural/remote areas and those with pregnancy complications during ANC [16]. In our previous studies [4, 5, 8, 9] we reported on the importance of ANC in promoting women’s utilisation of MWH and skilled facility delivery services. For example, during ANC, nurses and midwives inform pregnant women about the importance of giving birth at the health facility, and encourage those who live far from the health facilities and those with pregnancy complications to come to the health facility early and stay in the MWH. In the current study, we did not find a significant association between MWH utilisation and women’s age, parity, marital status, or socioeconomic status. This finding contradicts our previous studies [4, 5, 8, 9] that reported the importance of women’s sociodemographic characteristics in influencing their utilisation of MWHs. The reasons for this contradiction are not clear. Longitudinal studies are needed to test this association.

### Limitations of the study

Several potential limitations should be noted. First, these findings are based on responses from the women who accepted to be interviewed, and in that sense may suffer from response bias. Indeed, our sample ended up with most respondents (79.7%) who had given birth at the health centre, which could have been a sampling error as no women refused to be interviewed. It could have also been due to social desirability bias as it is possible that women who gave birth at home reported that they delivered at the health centre. Moreover, our results were positively skewed and we could not use parametric tests (such as Pearson’s correlation or multiple regression analysis) to investigate the associations of psychosocial measures with behavioural intention. Further, we do not have information on the response rate as it was difficult to find or systematically document information on the housing units in the research areas. Second, most respondents (79.7%) had given birth at the clinic; only 17% had given birth at home. This may have introduced a selection bias since the experiences of many women who had given birth at home were not explored. Moreover, selection of the respondents who were familiar with MWHs and maternal healthcare services in general, might have led to the severe skewness of the responses that we experienced on the psychosocial measures. Finally, since this is a cross-sectional study, it may not have the power to detect the effectiveness of MWHs in reducing the second phase delay and improving utilisation of maternal healthcare services. Furthermore, the study may not have the power to detect the contribution of MWHs to early detection and management of maternal and newborn health complications. To draw such a conclusion, future research should focus on longitudinal and quasi-experimental study designs.

## Conclusion

Our findings provide insight into the factors which are associated with pregnant women’s use of MWHs, including long distances to health facilities, number of ANC visits and history of pregnancy complications reported during ANC. These results can serve as a basis for the design of educational and public health interventions focusing on improving the utilisation of MWHs and access to facility-based skilled birth attendance. In particular, interventions should focus on: 1) promoting ANC use among pregnant women; 2) raising awareness about the risk and severity of pregnancy and labour complications; 3) promoting family and community support; 4) improving the quality of care by healthcare workers; 5) improving living conditions in the MWHs, and 6) mitigating logistical barriers that prevent women from using MWHs.

## Abbreviations

ANC: Antenatal Care
aOR: Adjusted Odds Ratio
LMICs: Low and middle income countries
MMR: Maternal Mortality Ratio
MOH: Ministry of Health
MWHs: Maternity Waiting Homes
NHC: Neighbourhood Health Committees
NS: Not Significant
OR: Odds Ratio
PBC: Perceived Behavioural Control
SD: Standard Deviation
WHO: World Health Organisation
